# Income Status and Life Satisfaction

**DOI:** 10.1007/s10902-021-00397-y

**Published:** 2021-05-11

**Authors:** Felix R. FitzRoy, Michael A. Nolan

**Affiliations:** 1 University of St Andrews, St Andrews, UK; 2 University of Hull, Cottingham Road, Hull, HU6 7RX UK

**Keywords:** Life satisfaction, Income rank, Relative income, I31

## Abstract

The importance of both income rank and relative income, as indicators of status, has long been recognised in the literature on life satisfaction and happiness. Recently, several authors have made explicit comparisons of the relative importance of these two measures of income status, and concluded that rank dominates to the extent that reference income becomes insignificant in regressions including both these explanatory variables, and that even absolute or household income, otherwise always positively related to happiness, may lose statistical significance. Here we test this hypothesis with a large UK panel (British Household Panel Survey and Understanding Society) for 1996–2017, split by age and retirement status, and find, contrary to previous results, that rank, household income and reference income are all usually important explanatory variables, but with significant differences between subgroups. This finding holds when rank is in its often-used relative form, and also with absolute rank.

## Introduction

The first large scale study of income rank as a determinant of overall life satisfaction (LS) is due to Boyce et al. (2010), using BHPS data, who found highly significant effects. Relative or comparison income, defined as the log mean of incomes in the reference group had frequently been found to have a significant negative effect on LS at least since Clark and Oswald (1996), but was statistically insignificant when included in LS regressions alongside within-group rank (relative to group size) in Boyce et al. (2010). Similarly, log (equivalised) household income, usually positively and significantly related to LS, also turned out to be insignificant.

Macchia et al. (2020) used a large international sample to study the effect of inequality, measured by the income share of the top 1%, and income rank, on well-being, and report that: ‘*Consistent with *Boyce et al. (2010)*, the inclusion of income rank removed the effect of absolute income*…’, while the effect of rank on multiple evaluations of well-being was significantly greater in more unequal countries. The latter result is plausible and complements existing evidence on the negative well-being effects of inequality—see Powdthavee et al. (2017) and Wilkinson and Pickett (2009).

However, the ‘pure’ rank-income hypothesis implies that the poorest, most deprived households, would *not* benefit from redistributing higher incomes, say through more progressive taxation, to reduce inequality, if rank order remained unchanged. This is contradicted by extensive evidence as noted above. Alleviating poverty also raises well-being by the standard concavity assumption of decreasing marginal utility of consumption. Empirically, LS of the rich does not increase much with income, and in the US, where most male hourly wages have stagnated since the late 1970s, and growth has mainly benefitted the richest, average LS has actually declined (and billionaires pay lower average total tax rates than the working class—see Rojas (2019) and Saez and Zucman (2019). By contrast, the egalitarian Nordic economies with high tax rates for top incomes typically rank highest in international comparisons of happiness or LS, with much lower rates of child and adult poverty than the UK and US (Dorling & Koljonen, 2020).

Persistent poverty also has serious effects on the next generation. After nearly a decade of austerity and welfare cuts, 30% of children in the UK are living in poverty, the highest share in Western Europe. Ongoing welfare cuts, exacerbated by the effects of the Covid-19 crisis, will likely raise child poverty close to 40% by 2021. ‘*For almost one in every two children to be poor in twenty-first century Britain would not just be a disgrace, but a social calamity and an economic disaster rolled into one’,* according to the UN Special Rapporteur on extreme poverty in a scathing indictment of ‘ideological policy’ in Britain (Alston, 2019). Mental health problems are usually found to have the largest direct negative effect on child and adult well-being, but are also closely correlated with poverty, as noted by Clark et al. (2018).

Growing up in poverty has many long term, lasting negative effects on long term future, adult mental and physical health, employment and earnings, and child mortality is also increasing due to poverty. ‘*…11% of young people aged 15–19 years in the UK are living in severe material deprivation. This is the fourth highest rate in Europe, with a worsening trend over time. …Material deprivation is judged on whether families can afford a decent meal every second day and to go on holiday, and whether they can cope with sudden unexpected financial expenses.*’ (Taylor-Robinson et al., 2019). To claim that raising these lowest incomes without changing the ranking of individual households would not increase their current well-being is clearly implausible to say the least. Meanwhile, the long-term benefits of reducing child poverty, both subjective and material, may be almost incalculable as they accumulate over whole future life courses—see Clark et al. (2018).

The rank-income and reference income hypotheses have been used, as in Rojas (2019), to explain the Easterlin Paradox—the observed lack of a strong relationship between long term economic growth and average happiness in advanced economies, and the positive cross-sectional correlation between income and LS. However, it is generally accepted that other factors are also important. Growing inequality and declining social capital both reduce LS, and income explains only a small proportion of the cross-sectional variation in LS—as pointed out by Clark et al. (2018), and by Frijters et al. (2019).

Empirically, we also reject the ‘pure’ rank-income hypothesis, first using the same data set and very similar specifications as Boyce et al. (2010), and finding that both absolute and reference household income remain significant when rank is included. Furthermore, extending their data up to 2018 with the new ‘Understanding Society’ survey panel, yielded similar results. Following our earlier work—see FitzRoy et al. (2014)—we split the panel by age, and confirm the positive ‘tunnel effect’ of comparison in the younger group. Previous authors have used a relative rank measure, but absolute rank which reflects reference group size seems theoretically more plausible—being in the top decile of a large reference group should confer more status than a similar position in a small group with generally less income dispersion, and this measure dominates relative rank in some but not all cases.

## Data

The data are drawn from Waves 6–10 and 12–18 of the British Household Panel Survey,^1^ (BHPS), covering a period that runs from 1996/1997 to 2008/2009; and from the portions of Waves 2–8 of the section of the new Understanding Society^2^ longitudinal study (University of Essex, Institute for Social and Economic Research, 2018) that involve active, consenting former members of the BHPS sample, covering a period^3^ from 2010 to 11 to 2016–2017. LS data were not collected for BHPS Wave 11 (17,609 observations for 2001/02)—not just for the overall LS measure we focus on in this paper, but also for satisfaction measures related to particular domains such as income, health and job. Our regression analysis generates results for up to 207,907 observations across 27,262 individuals, with those cases where there are missing values, and the highest income outliers,^4^ excluded. The deliberate over-sampling of the smaller nations of the UK since Wave 9 must be noted—so that about half of the observations in the BHPS (and its Understanding Society extension) are from Scotland, Wales and Northern Ireland,^5^ compared to about a sixth in the overall population. To check robustness, we briefly investigate how similar our results look when reweighting of observations is used so that the sample closely matches the population composition of the four component nations of the UK, as it averages across the period 1996–2017.

Our primary focus is on LS, and the impact upon it of several income-related variables. Since the measure of overall LS that we are using is subjective, the question of measurement error arises—with the potential for consequent attenuation bias, as laid out by Krueger and Schkade (2008). However, larger sample sizes are indicated in that earlier work to be a likely effective mitigating factor in terms of the practical impact of measurement error, and we are fortunate that our panel dataset provides very substantial samples. Our (deflated) measure of own household income (for the month before interview) is included alongside comparison (peer group) income. One matter that might deserve further consideration in determining the effect of comparison income is the systematic negative correlation that can be generated through exclusion bias. The issue stems from excluding an individual from their peer group to calculate their comparison income, whilst also including that individual in the peer groups of some others within the sample (when calculating the comparison income of those others), see Caeyers and Fafchamps (2020). It has also been suggested, by Powdthavee and Stutzer (2014), that those with higher incomes are less concerned with income comparison than others: in unreported estimations, with several thresholds for equivalised income, there is no substantive evidence of such a variation in the impact of comparison income on life satisfaction.

The definition used here for comparison income follows that employed by FitzRoy et al. (2014) and by FitzRoy and Nolan (2020)—whereby comparison groups are defined by age bands (between 3 years younger and 6 years older), sex, education (two categories), region (three categories) and Wave. A key aspect of this paper is the individual’s ranking within their comparison group. Absolute ranking is defined by ordering within-group incomes so that the lowest income receives rank1, and the highest income receives rank *m* in a group with *m* members., and hence with the highest potential rank number for the top income in the largest group. Relative rank (*rr*) is then defined (similarly to Boyce et al., 2010) as follows:

$$rr = {\text{ (Absolute}}\,{\text{rank }}{-} \, 1{)}/({\text{Group}}\,{\text{size }}{-} \, 1)$$

This lies in the range [0,1], and *rr* is highest (*ceteris paribus*) when absolute rank and income are highest. The key variables (including age) are summarised in Table 1. Group size for income rank is included, because it turns out that the attrition of the sample through time causes a noticeable reduction in average group size, and thus also the average of absolute income rank, for later waves. This reduction is also naturally reflected more among the older age sub-samples (any given individual is older, if present in later waves).

**Table 1:** UK: BHPS, waves 6–10, 12–18; Understanding Society, waves 2–8

	(1)	(2)	(3)	(3a)	(3b)
	All	Aged < 45	Aged 45 +	Aged 45 +, (not retired)	Aged 45 +, (retired)
*Summary statistics*					
Life satisfaction (mean)	5.21	5.15	5.26	5.10	5.47
Life satisfaction (SD)	1.34	1.42	1.41	1.39	1.41
Age (mean)	46.49	30.68	61.69	54.14	71.69
Age (SD)	18.53	8.44	11.56	7.09	8.27
Household income (mean)	2737.51	3076.53	2411.56	3059.05	1552.95
Household income (SD)	1844.06	1808.48	1819.01	1966.24	1129.88
Comparison income (mean)	2756.60	3132.96	2394.75	2918.03	1700.86
Comparison income (SD)	881.27	615.54	944.42	834.35	554.04
Absolute income rank (mean)	101.75	110.17	93.66	104.71	79.00
Absolute income rank (SD)	80.86	84.17	76.67	79.46	70.16
Group size, income rank (mean)	201.30	217.68	185.55	198.85	167.91
Group size, income rank (SD)	97.41	97.09	95.10	92.73	95.35
Relative income rank (mean)	0.50	0.50	0.50	0.53	0.47
Relative income rank (SD)	0.50	0.29	0.29	0.29	0.28
Observations	207,907	101,909	105,998	60,428	45,570

The overall regression specification includes a full set of regional dummies (with Greater London set as the reference region), and we also control for (the ILO measure^6^ of) regional unemployment—which is not exclusively cyclical, of course—as well as regional simple average (all dwelling) house prices.^7^ It is sometimes split by age range (below 45 and 45 + , respectively) and might take one of the following forms, for a typical fixed effects regression:

1a $${LS}_{it}={\beta }_{0}^{*}+{\beta }_{1}^{*}ln{\overline{Y }}_{jt}+{\beta }_{2}^{*}ln{Y}_{it}+{\beta }_{3}^{*}rr{Y}_{it}+{\boldsymbol{\alpha }}^{*}{\varvec{X}}_{\text{it}}+{v}_{i}^{*}+{\varepsilon }_{it}^{*},$$

1b $${LS}_{it}={\beta }_{0}+{\beta }_{1}ln{\overline{Y }}_{jt}+{\beta }_{2}ln{Y}_{it}+{\beta }_{3}AR{Y}_{it}+{\beta }_{4}GSAR{Y}_{it}+\boldsymbol{\alpha }{\varvec{X}}_{\text{it}}+{v}_{i}+{\varepsilon }_{it},$$

where the *i* subscript indexes the individual, the *t* subscript indexes the wave of the panel data, and *j* denotes the reference group (regarding individual *i*) for comparison income $\left(\overline{Y }\right)$. A set of wave dummies is included (BHPS Wave 6 being set as the control group). It should be kept in mind that the interview year for a given wave may differ by one across individuals. Household income is denoted *Y*, whilst absolute within-group rank of household income is shown as *ARY*, group size for absolute rank of income as *GSARY* and the corresponding relative rank is denoted *rrY*. The ***X*** term denotes a vector of additional included controls, alongside an attendant vector of estimated coefficients $\boldsymbol{\alpha }$. The individual fixed effect is termed *v*, whilst *ε* is the remaining disturbance term.

Summary statistics for the control variables are shown in Appendix Table 5—noting that the specification of age in our later estimates of Eq. (1a) and Eq. (1b) actually utilises a series of age-grouping dummies, rather than the age quadratic depicted in the table. Of the controls, a number that appear regularly in the literature are not predetermined in general—although several (such as marital status, education and region) will exhibit only limited variation through time for a given individual. Labour market status may be very stable for some people, but not others. An individual’s health status may fluctuate somewhat more, so we do undertake some robustness checks on its inclusion—finding an unsurprising substantial effect on overall LS, with its key impact otherwise being a strong boost to the regression’s explanatory power. It is striking that retired individuals exhibit the highest LS, whilst unretired persons in the 45 + age group are less satisfied than the average individual aged under 45, although the 45 + age range has a higher LS average overall than the under 45 s. Unsurprisingly, the household income average for retired people is lower than that for the other groups. It is mildly intriguing to see that household income has a slightly higher average amongst the younger age range compared to non-retired older people. However, rudimentary equivalisation of the household income figures via a division by the square root of household size yields an unsurprising reversal of the inequality (younger households tending to be larger). For within-group income ranking, Table 1 shows a higher absolute income rank average for the younger age group—and an expected lower mean rank for retired people. Relative income rank does not differ between the two age ranges, when averages are shown to two decimal places. However, within the older age range, retired individuals typically have an inferior within-group relative rank (and unretired people exhibit a correspondingly higher average relative rank).

Given the length of the time dimension now, across the BHPS and Understanding Society, it may be worth a further quick note at least on indirect possible evidence of attrition. So, for example, Population Trends data suggests the UK population to be about 51% female. Table 5, on the other hand, provides a figure of 54–55%. Similarly, the UK population may really have an average age of about 40, whereas Table 5 indicates about 46½—and the numerical dominance of females tends to increase with advancing age. On the other hand, the mean household size of 2.8 reported in the table appears rather on the high side for the UK average (2.4)—and household size more typically falls as an individual’s age rises.

## Results

We begin by considering some results with parallels to Boyce et al. (2010), including relative income rank alongside own household income and comparison income. The first two columns of Table 2, especially, are based on fixed effects estimation of Eq. (1a). As in FitzRoy et al. (2014) and in FitzRoy and Nolan (2020), we follow Moulton (1990) in recognising the potential (cluster-related) effect of aggregate regressors on standard errors. We assume clustering at the level of the individual. Column (1ai) uses all available years of data from the BHPS and its continuation into Understanding Society, whereas (1aii) only extends as far as BHPS Wave 13 (2003/04). On the other hand, the last two columns omit the fixed effect term and are instead estimated via pooled OLS, with standard errors clustered by reference income. As such, the estimates in column (1aiv) give the closest parallel to the main results from Boyce et al. (2010).

**Table 2:** UK: BHPS, waves 6–10, 12–18; Understanding Society, waves 2–8

	(1ai)	(1aii)	(1aiii)	(1aiv)
	All	BHPS, waves 6–10, 12–13	All	BHPS, waves 6–10, 12–13
*Fixed effects (FE) and (pooled) OLS*	FE	FE	OLS	OLS
Log of household income	0.028*** (3.04)	0.031** (2.04)	0.038*** (4.02)	0.050*** (3.47)
Log of comparison income	− 0.066* (− 1.69)	− 0.050 (− 0.83)	− 0.064*** (− 2.66)	− 0.084** (− 2.49)
Relative (within-group) rank of household income	0.052** (2.28)	0.061* (1.66)	0.138*** (6.39)	0.129*** (3.76)
Log of household size	− 0.083*** (− 5.42)	− 0.101*** (− 4.19)	− 0.079*** (− 7.62)	− 0.047*** (− 2.92)
Observations	207,907	86,733	207,907	86,733
Number of persons	27,262	22,643	27,262	22,643
Within R-squared	0.0344	0.0298	0.1512	0.1630

Dependent variable: life-satisfaction. Controls for marital status (including cohabiting), children, health status, education, work status, time in panel, year of last interview, household size, house ownership type, age group, wave number, regions, regional unemployment and regional house prices are included. Standard errors clustered at the level of the individual (FE), or reference income (OLS), robust t-statistics in parentheses

***p < 0.01, **p < 0.05, *p < 0.1

However, our particular specification of the list of regressors, alongside our definition of the comparison groups, leads to the retention of statistical significance for the pooled OLS estimates on both own household income and comparison income (column (1aiv)).^8^ This is in contrast to the results reported by the earlier authors, although we do have evidence of some reduction in the size of the own income estimate especially (compare to column (1aiv) above with Appendix Table 6, column (4)). There is also some inflation of the own income estimate’s standard error, probably due to the strong positive correlation between own income and relative income rank.^9^ Unsurprisingly (given the usual negative relationship between coefficient standard errors and sample size), our retained significance finding remains when the sample period is lengthened in column (1aiii), and the first part especially—on own household income—persists for a switch to fixed effects estimation in the first two columns (this is again unlike the earlier paper, where fixed effects results were mentioned in a footnote).

To investigate a little further, we attempt some sort of parallel with one of the Boyce et al. (2010) reference group definitions—specifically, the one based on twelve age groupings. We also withdraw the health status controls, to reduce the explanatory power of the regression in line with the earlier authors. The pooled OLS results (see Appendix Table 7) are somewhat more in line with the rank income hypothesis—own income, in particular, appears to lose statistical significance (columns (2) and (1aiv)). Comparison income is only statistically significant at the 10% level when the shorter sample up to BHPS Wave 13 is utilised. However, that is also true when within group income rank is omitted (see Table 7, column (3)). Indeed, for the full sample to Understanding Society Wave 8, comparison income retains its statistical significance when own income and within group income rank are also included (Table 7, column (1aiii)).

Table 3 displays results for further individual-specific fixed effects estimation of specifications like Eq. (1b)—with Eq. (1a)’s relative income rank regressor replaced by a pairing of absolute income rank alongside a control for income rank group size. This is worth considering because there is at least a possibility that a high relative ranking may serve to give a greater boost to LS if it has been achieved in the context of a larger comparator group of competitors. Five sets of summary estimates are presented, focused only on household income measures (along with household size, since this provides some relevant context). The first column of results captures the full sample, across the entire age range.^10^ Columns (2) and (3) then show estimates when individuals are split according to their age—with the boundary at the 45^th^ birthday. The final two columns, (3a) and (3b), then split the older age group in accordance with whether their current economic status is retirement (3b) or not (3a). It seems rather likely that incomes may differ systematically by retirement status, and so too may the impact of income on LS, and also the relationship between income status comparisons and LS.

**Table 3:** UK: BHPS, wave s 6–10, 12–18; Understanding Society, waves 2–8

	(1)	(2)	(3)	(3a)	(3b)
	All	Aged < 45	Aged 45 +	Aged 45 +, (not retired)	Aged 45 +, (retired)
*Fixed effects*					
Log of household income	0.029***	0.041***	0.008	0.012	0.011
	(3.64)	(3.80)	(0.70)	(0.83)	(0.49)
Log of comparison income	− 0.070*	0.162**	− 0.245***	− 0.052	− 0.184**
	(− 1.78)	(2.49)	(− 4.50)	(− 0.63)	(− 2.12)
Absolute (within-group)	0.00024***	0.00032***	0.00026*	0.00036**	− 0.000003
rank of household income	(2.70)	(2.73)	(1.88)	(2.05)	(− 0.01)
Group size (for rank of	0.00003	− 0.00019	0.00013	− 0.000002	0.00004
household income)	(0.31)	(− 1.62)	(0.89)	(− 0.01)	(0.18)
Log of household size	− 0.083***	− 0.064***	− 0.128***	− 0.141***	− 0.024
	(− 5.46)	(− 3.38)	(− 4.65)	(− 4.61)	(− 0.39)
Observations	207,907	101,909	105,998	60,428	45,570
Number of persons	27,262	17,790	13,580	10,147	7,153
R-squared	0.0344	0.0402	0.0302	0.0280	0.0322

Dependent variable: life-satisfaction. Controls for marital status (including cohabiting), children, health status, education, work status, time in panel, year of last interview, household size, house ownership type, age group, wave number, regions, regional unemployment and regional house prices are included. Standard errors clustered at the level of the individual, robust t-statistics in parentheses

***p < 0.01, **p < 0.05, *p < 0.1

The results in column (1)—across the full age-range of observations—indicate a traditional positive relationship between own (deflated) income (at the household level) and LS. There is also a statistically weak negative relationship between comparison income and LS, and this is also a fairly typical finding. Since the focus of this paper is on the impact of within-group rank of household income on LS, it is interesting to see a statistically significant positive effect of absolute within-group rank performance on LS. Meanwhile, the group size control has no significant impact. With the income rank effect showing up alongside an own income effect, our results differ from those found by Boyce et al. (2010), instead being very similar to those shown in column (1ai) of Table 2. One further point to note is that the specification could be adjusted to replace the log of own income by an equivalised measure of logged own income—by dividing own income by the square root of household size and then taking the log. Via algebraic equivalence, this would generate an identical (equivalised) own income estimate, whilst the estimate on the log of household size would become less negative by an amount equal to half the own income estimate^11^: it would remain statistically significant in column (1).

Column (2) and column (3) again demonstrate a result found elsewhere regarding comparison income, for example as in FitzRoy et al. (2014) and in FitzRoy and Nolan (2020). Comparison income exhibits a positive link to LS for the younger age range (up to 45), in contrast to the standard negative link found for the older age range (45 +). This is often attributed to the “tunnel effect”, as initially introduced by Hirschman and Rothschild (1973). As for the slightly shorter panel in FitzRoy and Nolan (2020), own income is positively linked to LS for those aged up to 45 (and like the full age range), but not significantly so for individuals of 45 +. New findings, relating to within-group household income rank, begin with absolute rank showing up with a positive link to LS in column (2), for those aged under 45. From column (3), however, the effect of absolute rank is only weakly statistically significant for the sample of older (45 +) people. The closest that the income rank group size control estimate comes to statistical significance is for the younger age group—in this instance, with a negative sign (potentially attenuating the positive effect of a high absolute income rank). Meanwhile, columns (3a) and (3b) then demonstrate that the effect of absolute rank amongst the older age range is evident only for those who have not retired from work. Perhaps those who have retired are less inclined to care about how their ranking within their group is changing, or maybe they are also less aware of that information. A further nuance is added to the picture by a simultaneous finding—across columns (3a) and (3b)—that comparison income, by contrast, has a traditional significant negative link with LS only amongst the retired sub-group. The insignificance of comparison income for non-retired older people (3a), typically between 45 and 65, may be interpreted as the result of two age-dependant comparison effects. The positive tunnel effect disappears as career paths become more settled by middle age, and the negative relative income effect grows with age for precisely the same reason. This cancellation of the two effects among older working individuals does not seem to have been identified previously. It arises alongside a statistically significant effect of absolute (within group) income ranking.^12^

A brief discussion of observed results for the controls is justified. Although not the focus of this paper in themselves, it may be reassuring to note that the estimates for many of the controls typically take the sign that might be most expected, on the basis of a combination of simple intuition and works elsewhere in the literature. We must keep in mind that our panel fixed effects model uses the fixed effects to control for differences between individuals, so the reported estimates (see Table 9 in the appendix for details in the case of the full age-range of observations) focus on within-individual variation. On marital status, having a partner (regardless of whether or not legally married) tends to increase LS—as in, for example, Grover and Helliwell (2019). From a reference category of being unemployed, each of the other main categories of economic activity status improves LS, apart from long-term sickness or disability—and this is in line with the corresponding conditional means of LS. Compared to intermediate health status, good health assists LS, whereas poor health reduces it. Having at least some housing ownership stake increases LS, relative to renting. Number of children has no statistically significant effect, whilst having high education interacts negatively with the earlier waves of the data (up to Wave 13 (2003/04). The impact of the age group controls on LS tends principally to exhibit a U-shape, as seen elsewhere.

Pooled OLS estimates are shown in Table 4. As is well known, fixed effects estimation has an advantage over pooled OLS estimation, in that it controls for potential effects drawn from omitted explanatory variables. However, the focus of fixed effects estimation on within (individual) variation across time is also a limitation—since it can be of interest to know how differences in characteristics between individuals appear to influence LS. For this reason, there is some value in examining results for pooled OLS estimation of a specification like Eq. (1b), but omitting the fixed effects term.

**Table 4:** UK: BHPS, waves 6–10, 12–18; Understanding Society, waves 2–8

	(1)	(2)	(3)	(3a)	(3b)
	All	Aged < 45	Aged 45 +	Aged 45 +, (not retired)	Aged 45 +, (retired)
*Pooled OLS*					
Log of household income	0.043*** (5.58)	0.047*** (4.38)	0.037*** (3.40)	0.038*** (2.71)	0.023 (1.38)
Log of comparison income	− 0.057** (− 2.41)	0.103*** (2.83)	− 0.069** (− 2.14)	− 0.174*** (− 4.14)	0.084 (1.40)
Absolute (within-group) rank of household income	0.00065*** (8.00)	0.00092*** (9.59)	0.00033*** (2.75)	0.00064*** (4.44)	− 0.00009 (− 0.47)
Group size (for rank of household income)	0.000003 (0.05)	− 0.00029*** (− 3.16)	0.00037*** (3.68)	0.00035*** (2.68)	0.00039*** (2.62)
Log of household size	− 0.080*** (− 7.95)	− 0.029** (− 2.11)	− 0.115*** (− 7.29)	− 0.123*** (− 6.60)	− 0.045 (− 1.43)
Observations	207,907	101,909	105,998	60,428	45,570
Number of persons	27,262	17,790	13,580	10,147	7,153
R-squared	0.1515	0.1390	0.1635	0.1679	0.1284

Dependent variable: life-satisfaction. Controls for marital status (including cohabiting), children, health status, education, work status, time in panel, year of last interview, household size, house ownership type, age group, wave number, regions, regional unemployment and regional house prices are included. Standard errors clustered at the level of reference income, robust t-statistics in parentheses

***p < 0.01, **p < 0.05, *p < 0.1

Own household income now shows up with a positive and statistically significant effect on LS in columns (1)-(4), so its impact here is robust to splits by age, but not retirement status (see column (5)). It should also be noted that a switch to equivalised own household income (via algebraic equivalence) would leave a significant negative remaining effect of household size in all instances except the younger age sub-group (column (2)) and the retired sub-group (column (5)). The effects of comparison income again support the existence of the ‘tunnel effect’, but the split of the older group by retirement status in Table 4 shows a significant negative estimate for those who have not retired and a statistically insignificant positive estimate for those who have—so the latter especially is quite different from its (negative and significant) fixed effects counterpart. Column (4) also includes a significant positive effect for absolute income rank. Overall, pooled OLS results for absolute income ranking are just like their fixed effects counterparts—positive and statistically significant in the first four columns of the table, and again negative and statistically insignificant for the retired sub-group. However, it is interesting to see that the group size control estimates are now statistically significant at the 1% level, with only one exception. Notably, this exception occurs for the whole sample in column (1), and it is a consequence of the ‘cancelling out’ effect of a contrasting negative effect in the younger group (column (2)) and a corresponding positive effect for the older group (column (3)).^13^

The detailed set of estimates from column (1), across the full age-range, are laid out in Table 10 in the appendix. It is evident that pooled OLS generates larger t ratios for almost all regressors, usually with same sign for the estimate. Divorced status is one exception here—having a negative effect for the pooled OLS estimate (mirroring the lower raw LS mean), but a positive sign for fixed effects. This might suggest an improvement in LS for a given individual whose marital status switches (presumably for particular and non-random reasons) to being divorced. Another exception relates to having children—for which the pooled OLS estimates are negative and significant at the 10% level or better, again reflecting lower raw mean LS. Here, the fixed effects estimates are positive, but statistically insignificant. It is not surprising that, for a given individual, having one or more children does not seem to have a negative impact on LS: again, those who have children are not a random sample of the population.

In the interests of robustness verification, and given that LS is assigned to seven ordered categories, a check was made on the results from estimation of a random effects panel data ordered probit model. The positive sign of the own income estimate was again evident, and with statistical significance in the overall sample and the under 45 sub-sample only—as with fixed effects regression estimation. There was also evidence of the ‘tunnel’ effect for comparison income, with the ordered probit results being a variant of both the fixed effects results, and those from pooled OLS. For absolute income rank and the group size control, the ordered probit estimates were rather similar to the pooled OLS estimates^14^—rank positively influences LS, apart from when the older group is split by retirement status.

A further robustness check on the specification of the rank variable is offered by Tables 11 (fixed effects) and 12 (pooled OLS) in the appendix. Both the rank regressor, and its accompanying group size counterpart, are specified there in an alternative log-transformed form. The results for own income and (within-group) income rank are less statistically significant than those in the counterpart tables above (Tables 3 and 4). However, taking the log of an integer rank variable, and of an integer group size variable, are perhaps less obviously justified than using a log form for (non-integer) own income and comparison group income levels.

Given potential concerns about the inclusion of health status controls, which might readily be considered endogenous alongside the determination of LS, Tables 13 and 14 in the appendix show the key estimates when health status is omitted from the regression. Whilst there are inevitably some modest changes in the magnitude of the coefficients, the thrust of the signs and statistical significance of the estimates remains very similar. Probably unsurprisingly, the main change that can be seen is the rather substantial reduction in the explanatory power of each of the regressions.

It should be noted that the effects of both income and rank variables on LS are very small in magnitude, unsurprisingly and consistent with the usual finding that health, work, family and social relationships are the main determinants of LS. In spite of this, our main finding is perhaps more surprising: that the rank variables do generally attain high levels of statistical significance in the presence of the usual income variables.

## Conclusions

A few intriguing findings have emerged from our analysis of the impact of (within group) income rank on overall life satisfaction (LS) using UK panel data from the BHPS and its extension in Understanding Society. Across the whole sample, a positive link is found between income rank and LS (so a better ranking position gives greater satisfaction), but this does not result in the effects of the own income variable and relative income variable both becoming statistically insignificant, in contrast to the finding by Boyce et al. (2010). This contradiction of the earlier authors holds whether we try a relative income rank measure or our preferred absolute rank alternative. It also remains for the shorter sample period available to the earlier authors, as well as for the longer timespan of data now available. Whilst it is present for both fixed effects estimation and pooled OLS, the estimate for absolute (within group) income rank does lose statistical significance for the shorter sample in the fixed effects case. However, the general significance of rank in most specifications does suggest that the exclusion of this variable from most existing studies of LS or happiness may have resulted in omitted variable bias.

Our split of the sample by age confirms previous comparison income findings, for example as in FitzRoy et al. (2014) and in FitzRoy and Nolan (2020) for shorter panels of UK data. A negative effect of comparison income across the whole age range masks an opposing sign for the ‘tunnel effect’ of comparison income amongst the under 45 s. The only departure from statistical significance at the 5% level, across both fixed effects estimation and pooled OLS, is for the overall sample under fixed effects (instead, only significant at the 10% level). In this paper, of course, the findings have also included control for (within group) income rank—which was not the case in the earlier articles. Non-retired over 45 s are shown to have broadly similar results to all over 45 s, except that the estimate for (within group) absolute income rank was smaller in size for the non-retired sub-group under both estimation methods. Also, the negative fixed effects estimate for comparison income was much smaller (and insignificant)—unlike the retired sub-group’s estimate, itself a contrast to the retired sub-group’s many insignificant results for both estimation methods.

From a policy perspective, our finding that a pure income rank hypothesis does not appear to hold would appear to leave room for policy-makers that care about LS to consider some redistributive measures to simultaneously reduce income inequality and have a positive influence on aggregate LS. Not only that, but there may be scope to tailor such policies specifically to suit the distinct typical LS tastes of older and younger people. The effectiveness of such a policy agenda could be further enhanced by future research to investigate the evidence on the correlates of narrower measures of LS—based on specific domains such as income, health or leisure time. Many domains are likely to contribute to overall LS, and there is an already recognised issue of principle about how these domains should be weighted.

## Data Availability

See “Acknowledgements” on data sources. Some supplementary material regarding results can be made available, if desired (over and above the current appendix).

## Appendix

See Tables 5, 6, 7, 8, 9, 10, 11, 12, 13 and 14.

**Table 5﻿:** UK: BHPS, waves 6–10, 12–18; Understanding Society, waves 2–8

	(1)	(2)	(3)	(3a)	(3b)
	All	Aged < 45	Aged 45 +	Aged 45 +, not retired	Aged 45 +, retired
*Sample means*					
Life satisfaction	5.209	5.151	5.264	5.104	5.477
Female	0.547	0.542	0.553	0.532	0.580
Age	46.489	30.678	61.690	54.143	71.696
Age squared (divided by 50)	50.091	20.248	78.783	59.634	104.175
Last wave	0.115	0.124	0.106	0.099	0.116
Run of waves	8.891	6.936	10.770	10.557	11.053
Run of waves squared	114.712	72.794	155.014	149.528	162.289
Household size	2.842	3.393	2.313	2.701	1.798
Employee	0.505	0.648	0.367	0.643	0.000
Self-employed	0.069	0.065	0.072	0.126	0.000
Retired	0.219	0.001	0.430	0.000	1.000
Family care or maternity	0.068	0.086	0.051	0.090	0.000
Full-time study	0.057	0.116	0.001	0.002	0.000
Long-term sick or disabled	0.041	0.026	0.056	0.098	0.000
Other economic activity	0.006	0.008	0.005	0.008	0.000
Unemployed	0.035	0.051	0.019	0.034	0.000
Invalidity benefit	0.012	0.008	0.015	0.016	0.014
Married	0.532	0.407	0.652	0.704	0.584
Cohabiting	0.113	0.179	0.049	0.070	0.021
Widowed	0.071	0.003	0.137	0.040	0.265
Divorced	0.058	0.033	0.082	0.098	0.061
Health positive	0.629	0.714	0.547	0.599	0.478
Health negative	0.122	0.075	0.168	0.142	0.203
Low educated	0.539	0.463	0.613	0.553	0.693
Medium educated	0.311	0.360	0.264	0.292	0.226
Highly educated	0.150	0.177	0.123	0.155	0.081
One child	0.120	0.177	0.065	0.112	0.003
Two children	0.114	0.198	0.033	0.058	0.001
Three or more children	0.046	0.085	0.009	0.016	0.0002
Own house outright	0.288	0.103	0.465	0.326	0.649
Mortgaged house	0.440	0.584	0.302	0.462	0.090
Regional ILO unemployment rate	5.979	5.971	5.987	5.982	5.995
Regional house price	150,179.2	145,991.2	154,205.6	154,656.0	153,608.4
Greater London	0.049	0.052	0.047	0.051	0.042
South East	0.094	0.097	0.091	0.095	0.085
South West	0.063	0.060	0.065	0.066	0.063
East Anglia	0.063	0.061	0.065	0.065	0.064
East Midlands	0.059	0.065	0.053	0.057	0.047
West Midlands	0.057	0.059	0.055	0.055	0.055
Yorkshire and the Humber	0.062	0.065	0.060	0.057	0.064
North East	0.029	0.030	0.029	0.029	0.029
North West	0.081	0.083	0.079	0.079	0.078
Wales	0.158	0.146	0.170	0.153	0.192
Scotland	0.171	0.170	0.172	0.171	0.174
Northern Ireland	0.114	0.112	0.116	0.123	0.108
Observations	207,907	101,909	105,998	60,428	45,570

**Table 6:** UK: BHPS, waves 6–10, 12–18; Understanding Society, waves 2–8

	(1)	(2)	(3)	(4)
	All	BHPS, waves 6–10, 12–13	All	BHPS, waves 6–10, 12–13
*Fixed effects (FE) and (pooled) OLS*	FE	FE	OLS	OLS
Log of household income	0.044***	0.048***	0.090***	0.097***
	(7.14)	(5.14)	(16.77)	(12.11)
Log of comparison income	− 0.072*	− 0.056	− 0.080***	− 0.094***
	(− 1.86)	(− 0.92)	(− 3.35)	(− 2.76)
Log of household size	− 0.089***	− 0.107***	− 0.103***	− 0.068***
	(− 5.91)	(− 4.56)	(− 10.60)	(− 4.56)
Observations	207,907	86,733	207,907	86,733
Number of persons	27,262	22,643	27,262	22,643
Within R-squared	0.0343	0.0298	0.1510	0.1628

Dependent variable: life-satisfaction. Controls for marital status (including cohabiting), children, health status, education, work status, time in panel, year of last interview, household size, house ownership type, age group, wave number, regions, regional unemployment and regional house prices are included. Standard errors clustered at the level of the individual (FE), or reference income (OLS), robust t-statistics in parentheses

****p* < 0.01, ***p* < 0.05, **p* < 0.1

**Table 7:** UK: BHPS, waves 6–10, 12–18; Understanding Society, waves 2–8

	(1aiii)	(2)	(3)	(1aiv)
	All	BHPS, waves 6–10, 12–13	BHPS, waves 6–10, 12–13	BHPS, waves 6–10, 12–13
*(Pooled) OLS*	OLS	OLS	OLS	OLS
Log of household income	0.004	0.030	0.116***	0.031
	(0.38)	(1.54)	(10.97)	(1.62)
Log of comparison income	− 0.111***		− 0.070*	− 0.067*
	(− 4.01)		(− 1.78)	(− 1.77)
Relative (within-group) rank of	0.921***	0.747***		0.745***
household income	(8.67)	(4.13)		(4.14)
Log of household size	− 0.077***	− 0.058***	− 0.095***	− 0.057***
	(− 6.26)	(− 2.92)	(− 5.15)	(− 2.87)
Observations	207,907	86,733	86,733	86,733
Number of persons	27,262	22,643	22,643	22,643
Within R-squared	0.0853	0.0922	0.0917	0.0922

Dependent variable: life-satisfaction. Controls for marital status (including cohabiting), children, education, work status, time in panel, year of last interview, household size, house ownership type, age group, wave number, regions, regional unemployment and regional house prices are included. Standard errors clustered at the level of reference income (OLS), robust t-statistics in parentheses. Reference income groups (for each wave) are based on the same 12 age groupings as those used by Boyce et al. (2010)

****p* < 0.01, ***p* < 0.05, **p* < 0.1

**Table 8:** UK: BHPS, waves 6–10, 12–18; Understanding Society, waves 2–8

	(1ai)	(1aii)	(1aiii)	(1aiv)
	All	BHPS, waves 6–10, 12–13	All	BHPS, waves 6–10, 12–13
*Fixed effects (FE) and (pooled) OLS*	FE	FE	OLS	OLS
Log of household income	0.029***	0.037***	0.043***	0.057***
	(3.64)	(2.90)	(5.58)	(4.80)
Log of comparison income	− 0.070*	− 0.050	− 0.057**	− 0.064*
	(− 1.78)	(− 0.83)	(− 2.41)	(− 1.86)
Absolute (within-group)	0.00024***	0.00019	0.00065***	0.00053***
rank of household income	(2.70)	(1.46)	(8.00)	(4.17)
Group size (for rank of	0.00003	− 0.00004	0.000003	0.00004
household income)	(0.31)	(− 0.31)	(0.05)	(0.45)
Log of household size	− 0.083***	− 0.103***	− 0.080***	− 0.048***
	(− 5.46)	(− 4.32)	(− 7.95)	(− 3.11)
Observations	207,907	86,733	207,907	86,733
Number of persons	27,262	22,643	27,262	22,643
Within R-squared	0.0344	0.0298	0.1515	0.1632

Dependent variable: life-satisfaction. Controls for marital status (including cohabiting), children, health status, education, work status, time in panel, year of last interview, household size, house ownership type, age group, wave number, regions, regional unemployment and regional house prices are included. Standard errors clustered at the level of the individual (FE), or reference income (OLS), robust t-statistics in parentheses

****p* < 0.01, ***p* < 0.05, **p* < 0.1

**Table 9:** Individual fixed effects, whole age range—UK: BHPS, waves 6–10, 12–18; Understanding Society, waves 2–8

Regressor	Estimate	T ratio	Regressor	Estimate	T ratio
Aged 25–34	− 0.006	− 0.36	Employee	0.266***	12.72
Aged 35–44	− 0.049*	− 1.91	Self-employed	0.263***	9.91
Aged 45–54	− 0.038	− 1.17	Retired	0.343***	13.35
Aged 55–64	0.074*	1.91	Family care or maternity leave	0.247***	9.95
Aged 65–74	0.131***	2.77	Full-time study	0.385***	14.35
Aged 75 +	0.085	1.54	Long-term sickness or disabled	− 0.143***	− 4.51
Last wave	− 0.091***	− 8.17	Other economic activity	0.241***	6.12
Run of waves	− 0.010***	− 3.64	Invalidity benefit	− 0.040	− 1.13
Run of waves squared	0.00078***	7.11	Health status positive	0.212***	27.67
Comparison income (log form)	− 0.070*	− 1.78	Health status negative	− 0.336***	− 25.88
Household income (log form)	0.029***	3.64	Medium education	0.004	0.21
Log of household size	− 0.083***	− 5.46	High education	− 0.082**	− 2.43
Rank of household income (within group)	0.00024***	2.70	High education * Wave 14 +	0.033	1.06
Group size for household income ranking	0.00003	0.31	One child	0.015	1.05
			Two children	0.006	0.33
			Three or more children	0.019	0.73
			Own house (outright)	0.063***	4.35
Married	0.215***	10.95	Owner occupier with mortgage	0.029**	2.30
Cohabiting	0.221***	12.72	Log of regional house price	− 0.038	− 0.95
Widowed	− 0.017	− 0.44	ILO unemployment rate (region)	− 0.010	− 1.54
Divorced	0.059**	2.05	Female	1.659	0.95
Region dummies	11		Rho	0.622	
Wave dummies	18		R-squared within	0.0344	
Observations	207,307		R-squared between	0.0121	
Individuals	27,262		R-squared overall	0.0123	
sigma_u, sigma e	1.311	1.021	Correlation(u_i, Xb)	− 0.6137	

Dependent variable: life-satisfaction

****p* < 0.01, ***p* < 0.05, **p* < 0.1

**Table 10:** Pooled OLS, whole age range—UK: BHPS, waves 6–10, 12–18; Understanding Society, waves 2–8

Regressor	Estimate	T ratio	Regressor	Estimate	T ratio
Aged 25–34	− 0.191***	− 14.20	Employee	0.346***	18.41
Aged 35–44	− 0.320***	− 21.84	Self-employed	0.378***	17.92
Aged 45–54	− 0.295***	− 19.71	Retired	0.485***	21.79
Aged 55–64	− 0.107***	− 6.25	Family care or maternity leave	0.338***	15.43
Aged 65–74	0.065**	2.45	Full-time study	0.534***	23.76
Aged 75 +	0.144***	5.13	Long-term sickness or disabled	− 0.170***	− 6.52
Last wave	− 0.102***	− 9.52	Other economic activity	0.328***	7.90
Run of waves	− 0.009***	− 4.77	Invalidity benefit	− 0.219***	− 7.24
Run of waves squared	0.00019**	2.19	Health status positive	0.498***	68.20
Comparison income (log form)	− 0.057**	− 2.41	Health status negative	− 0.644***	− 51.63
Household income (log form)	0.043***	5.58	Medium education	− 0.022***	− 2.64
Log of household size	− 0.080***	− 7.95	High education	− 0.121***	− 8.28
Rank of household income (within group)	0.00065***	8.00	High education * Wave 14 +	0.017	1.39
Group size for household income ranking	0.000003	0.05	One child	− 0.044***	− 4.33
			Two children	− 0.019*	− 1.67
			Three or more children	− 0.028*	− 1.70
			Own house (outright)	0.126***	14.04
Married	0.349***	35.06	Owner occupier with mortgage	0.063***	7.95
Cohabiting	0.259***	23.86	Log of regional house price	− 0.017	− 0.45
Widowed	0.112***	6.41	ILO unemployment rate (region)	− 0.004	− 0.62
Divorced	− 0.073***	− 4.81	Female	0.007	1.01
Region Dummies	11				
Wave Dummies	18		R-squared	0.1515	
Observations	207,307				
Individuals	27,262				

Dependent variable: life-satisfaction

****p* < 0.01, ***p* < 0.05, **p* < 0.1

**Table 11:** UK: BHPS, wave s 6–10, 12–18; Understanding Society, waves 2–8

	(1)	(2)	(3)	(3a)	(3b)
	All	Aged < 45	Aged 45 +	Aged 45 +, (not retired)	Aged 45 +, (retired)
*Fixed effects*					
Log of household income	0.018 (1.61)	0.024 (1.52)	0.004 (0.25)	0.004 (0.18)	− 0.001 (− 0.03)
Log of comparison income	− 0.068* (− 1.74)	0.170*** (2.62)	− 0.238*** (− 4.35)	− 0.052 (− 0.61)	− 0.178** (− 2.05)
Log of absolute (within-group) rank of household income	0.022*** (2.70)	0.031*** (2.60)	0.016 (1.49)	0.025 (1.60)	0.008 (0.49)
Log of group size (for rank of household income)	0.011 (0.73)	− 0.027 (− 1.26)	0.017 (0.76)	0.012 (0.39)	− 0.012 (− 0.35)
Log of household size	− 0.077*** (− 4.93)	− 0.056*** (− 2.81)	− 0.124*** (− 4.43)	− 0.135*** (− 4.33)	− 0.019 (− 0.30)
Observations	207,907	101,909	105,998	60,428	45,570
Number of persons	27,262	17,790	13,580	10,147	7,153
R-squared	0.0344	0.0402	0.0301	0.0279	0.0322

Dependent variable: life-satisfaction. Controls for marital status (including cohabiting), children, health status, education, work status, time in panel, year of last interview, household size, house ownership type, age group, wave number, regions, regional unemployment and regional house prices are included. Standard errors clustered at the level of the individual, robust t-statistics in parentheses

****p* < 0.01, ***p* < 0.05, **p* < 0.1

**Table 12:** UK: BHPS, waves 6–10, 12–18; Understanding Society, waves 2–8

	(1)	(2)	(3)	(3a)	(3b)
	All	Aged < 45	Aged 45 +	Aged 45 +, (not retired)	Aged 45 +, (retired)
*Pooled OLS*					
Log of household income	0.053*** (4.92)	0.057*** (3.46)	0.036** (2.46)	0.012 (0.63)	0.007 (0.28)
Log of comparison income	− 0.064*** (− 2.64)	0.099*** (2.71)	− 0.069** (− 2.10)	− 0.169*** (− 4.02)	0.088 (1.50)
Log of absolute (within-group) rank of household income	0.029*** (3.75)	0.048*** (4.13)	0.019* (1.88)	0.059*** (3.97)	0.007 (0.48)
Log of group size (for rank of household income)	0.024** (2.13)	− 0.016 (− 0.89)	0.052*** (3.39)	0.042* (1.88)	0.056** (2.31)
Log of household size	− 0.086*** (− 8.04)	− 0.040*** (− 2.66)	− 0.114*** (− 6.83)	− 0.113*** (− 5.73)	− 0.038 (− 1.10)
Observations	207,907	101,909	105,998	60,428	45,570
Number of persons	27,262	17,790	13,580	10,147	7,153
R-squared	0.1512	0.1383	0.1633	0.1677	0.1285

Dependent variable: life-satisfaction. Controls for marital status (including cohabiting), children, health status, education, work status, time in panel, year of last interview, household size, house ownership type, age group, wave number, regions, regional unemployment and regional house prices are included. Standard errors clustered at the level of reference income, robust t-statistics in parentheses

****p* < 0.01, ***p* < 0.05, **p* < 0.1

**Table 13:** UK: BHPS, Waves 6–10, 12–18; Understanding Society, Waves 2–8

	(1)	(2)	(3)	(3a)	(3b)
	All	Aged < 45	Aged 45 +	Aged 45 +, (not retired)	Aged 45 +, (retired)
*Fixed effects*					
Log of household income	0.030*** (3.71)	0.044*** (3.99)	0.008 (0.67)	0.012 (0.78)	0.011 (0.50)
Log of comparison income	− 0.104*** (− 2.63)	0.175*** (2.67)	− 0.306*** (− 5.57)	− 0.056 (− 0.67)	− 0.229*** (− 2.62)
Absolute (within-group) rank of household income	0.00023** (2.49)	0.00032*** (2.67)	0.00023 (1.62)	0.00036** (2.01)	− 0.00004 (− 0.15)
Group size (for rank of household income)	0.000003 (0.04)	− 0.00017 (1.46)	0.00015 (1.04)	− 0.000006 (− 0.03)	0.00009 (0.39)
Log of household size	− 0.082*** (− 5.36)	− 0.068*** (− 3.51)	− 0.133*** (− 4.79)	− 0.143*** (− 4.67)	− 0.028 (− 0.45)
Observations	207,907	101,909	105,998	60,428	45,570
Number of persons	27,262	17,790	13,580	10,147	7,153
R-squared	0.0195	0.0232	0.0180	0.0181	0.0203

Dependent variable: life-satisfaction. Controls for marital status (including cohabiting), children, education, work status, time in panel, year of last interview, household size, house ownership type, age group, wave number, regions, regional unemployment and regional house prices are included. Standard errors clustered at the level of the individual, robust t-statistics in parentheses

****p* < 0.01, ***p* < 0.05, **p* < 0.1

**Table 14:** UK: BHPS, waves 6–10, 12–18; Understanding Society, waves 2–8

	(1)	(2)	(3)	(3a)	(3b)
	All	Aged < 45	Aged 45 +	Aged 45 +, (not retired)	Aged 45 +, (retired)
*Pooled OLS*					
Log of household income	0.055*** (6.73)	0.058*** (5.23)	0.051*** (4.34)	0.049*** (3.34)	0.051*** (2.68)
Log of comparison income	− 0.107*** (− 4.31)	0.125*** (3.30)	− 0.115*** (− 3.43)	− 0.185*** (− 4.24)	0.073 (1.18)
Absolute (within-group) rank of household income	0.00077*** (8.42)	0.00107*** (10.66)	0.00040*** (2.84)	0.00083*** (5.56)	− 0.00023 (− 1.05)
Group size (for rank of household income)	− 0.00022*** (− 2.95)	− 0.00037*** (− 3.80)	0.00026** (2.50)	0.00027* (1.96)	0.00048*** (3.01)
Log of household size	− 0.093*** (− 8.89)	− 0.040*** (− 2.79)	− 0.134*** (− 8.35)	− 0.131*** (− 6.78)	− 0.103*** (− 3.20)
Observations	207,907	101,909	105,998	60,428	45,570
Number of persons	27,262	17,790	13,580	10,147	7,153
R-squared	0.0858	0.0827	0.0917	0.1140	0.0315

Dependent variable: life-satisfaction. Controls for marital status (including cohabiting), children, education, work status, time in panel, year of last interview, household size, house ownership type, age group, wave number, regions, regional unemployment and regional house prices are included. Standard errors clustered at the level of reference income, robust t-statistics in parentheses

****p* < 0.01, ***p* < 0.05, **p* < 0.1

## Footnotes

1 The earlier waves of the BHPS (up to Wave 10) were limited in coverage to Great Britain. The full United Kingdom (including Northern Ireland) is covered in Waves 12–18. BHPS data are available via the UK Data Service (formerly the UK Data Archive).

2 Since Wave 2 of Understanding Society is the first to follow on from BHPS Wave 18, we essentially treat the Understanding Society waves (2–8) as 19–25.

3 With interviews taking place across calendar year boundaries (and two boundaries for Waves 21–22), a given Wave will see certain regressors defined according the year of interview, as appropriate to each individual.

4 A cut-off of 9.5 for the natural logarithm of (deflated) monthly household income is around £160,000 per year. This reduces the number of observations by 684, whilst a further 19 cases are excluded due to issues relating to the identification of individuals across waves. By definition, regressors measuring changes in household income between successive waves are not available for the first wave in which any individual responds. This is some 18,010 observations.

5 Across Waves 6–25, 44% of observations are for individuals outside England – whereas the population percentage is closer to 16–17%. Northern Ireland was not included in the BHPS data until Wave 11 (2001/02).

6 The annual ILO unemployment rates for NUTS1 regions of the UK are to be found in series YCNC-YCNK and YCNM-YCNN.

7 The use of a simple average of house prices across all dwellings is a simplification, but it does enable the availability of a longer continuous run of data. There might be two opposing components of regional house prices on happiness – for some, higher house prices are for an owned asset; for others, they indicate lower affordability of their location.

8 These findings are just the same if the sample is reweighted to reflect the actual population composition of the UK across its four constituent nations. These estimates are available from the authors, on request.

9 This correlation is near to + 0.73 for both the longer and shorter samples. By contrast, there is little change in the comparison income estimate’s standard error – in line with the near orthogonality found here for comparison income and (within-group) income rank (a correlation of around + 0.03 in each sample).

10 Further context is offered by Table 8 in the appendix—whose estimates are for specifications which mirror those underlying Table 2, but with absolute income ranking (and group size) replacing relative income rank. Overall, the estimates in Tables 2 and 8 are broadly similar.

11 Alongside equivalized own income, the estimate on the log of household size would be − 0.068, with a t ratio of − 4.79.

12 Unreported results do not find similar statistical significance for relative income rank, if that is used instead.

13 It should be noted that the group size control is not statistically significant for the 45 + age group (columns (3), (3a) and (3b)) when the sample is reweighted to reflect UK population composition amongst its four constituent nations.

14 Of course, the ordered probit model does enable the calculation of a substantially richer structure of marginal effects for the probability of an individual being in each of the seven LS categories. However, in the panel context, the calculations required to produce these marginal effects and their standard errors are rather time-consuming. Nor can the marginal effects be easily reported in an abbreviated form, so we refrain from taking this robustness check to its fullest extent.

## Code availability

The estimates and summary statistics were generated using Stata 16 (econometric software from StataCorp).

## References

[CR1] Alston, P. (2019). Visit to the United Kingdom of Great Britain and Northern Ireland. https://undocs.org/A/HRC/41/39/Add.1.

[CR2] Boyce CJ, Brown GDA, Moore SC (2010). Money and happiness: Rank of income, not income, affects life satisfaction. Psychological Science.

[CR3] Caeyers, B., & Fafchamps, M. (2020). *Exclusion bias in the presence of peer effects*. CEPR discussion paper no. DP14386, Available at SSRN: https://ssrn.com/abstract=3535472.

[CR4] Clark, A. E., Flèche, S., Layard, R., Powdthavee, N., & Ward, G. (2018). *The origins of happiness*. Princeton University Press.

[CR5] Clark A, Oswald A (1996). Satisfaction and comparison income. Journal of Public Economics.

[CR6] Dorling, D., & Koljonen, A. (2020). *Finntopia*. Agenda Publishing.

[CR7] FitzRoy FR, Nolan MA, Steinhardt MF, Ulph D (2014). Testing the tunnel effect: Comparison, age and happiness UK and German panels. IZA Journal of European Labor Studies.

[CR8] FitzRoy FR, Nolan MA (2020). Education, income and happiness: Panel evidence for the UK. Empirical Economics.

[CR9] Frijters, P., Clark, A. E., Krekel, C., & Layard, R. (2019). *A happy choice: Wellbeing as the goal of government*. CEP discussion paper no 1658, October 2019.

[CR10] Grover S, Helliwell JF (2019). How’s life at home? New evidence on marriage and the set point for happiness. Journal of Happiness Studies.

[CR11] Hirschman AO, Rothschild M (1973). The changing tolerance for income inequality in the course of economic development. The Quarterly Journal of Economics.

[CR12] Krueger AB, Schkade DA (2008). The reliability of subject well-being measures. Journal of Public Economics.

[CR13] Macchia L, Plagnol A, Powdthavee N (2020). Buying happiness in an unequal world: Rank of income more strongly predicts well-being in more unequal countries. Personality and Social Psychology Bulletin.

[CR14] Moulton BR (1990). An illustration of a pitfall in estimating the effects of aggregate variables on micro units. Review of Economics and Statistics.

[CR15] Powdthavee N, Burkhauser RV, De Neve JE (2017). Top incomes and human well-being: Evidence from the Gallup World Poll. Journal of Economic Psychology.

[CR16] Powdthavee, N., & Stutzer, A. (2014). *Economic approaches to understanding change in happiness*. IZA discussion paper no. 8131.

[CR17] Rojas, M. (2019). *The economics of happiness*. Springer.

[CR18] Saez, E., & Zucman, G. (2019). How would a progressive wealth tax work? Evidence from the economics literature. *Brookings Papers on Economic Activity Conference Drafts*.

[CR19] Taylor-Robinson D, Lai ETC, Wickham S, Rose T, Norman P, Bambra C, Whitehead MT, Barr BN (2019). Assessing the impact of rising child poverty on the unprecedented rise in infant mortality in England, 2000–2017: Time trend analysis. British Medical Journal Open.

[CR20] University of Essex, Institute for Social and Economic Research. (2018). *Understanding society: Waves 1–8, 2009–2017 and harmonised BHPS: Waves 1–18, 1991–2009*. [data collection]. 11th Edition. UK Data Service. SN: 6614. 10.5255/UKDA-SN-6614-13.

[CR21] Wilkinson, R., & Pickett, K. (2009). *The spirit level*. Allen Lane.

